# Room-temperature synthesis of nanoporous 1D microrods of graphitic carbon nitride (g-C_3_N_4_) with highly enhanced photocatalytic activity and stability

**DOI:** 10.1038/srep31147

**Published:** 2016-08-08

**Authors:** Rajendra C. Pawar, Suhee Kang, Jung Hyun Park, Jong-ho Kim, Sunghoon Ahn, Caroline S. Lee

**Affiliations:** 1 Department of Materials Engineering,Hanyang University, 426-791, South Korea; 2 Department of Chemical Engineering, Hanyang University, 426-791, South Korea; 3 School of Mechanical & Aerospace Engineering,Seoul National University, 151-742, South Korea

## Abstract

A one-dimensional (1D) nanostructure having a porous network is an exceptional photocatalytic material to generate hydrogen (H_2_) and decontaminate wastewater using solar energy. In this report, we synthesized nanoporous 1D microrods of graphitic carbon nitride (g-C_3_N_4_) via a facile and template-free chemical approach at room temperature. The use of concentrated acids induced etching and lift-off because of strong oxidation and protonation. Compared with the bulk g-C_3_N_4_, the porous 1D microrod structure showed five times higher photocatalytic degradation performance toward methylene blue dye (MB) under visible light irradiation. The photocatalytic H_2_ evolution of the 1D nanostructure (34 μmol g^−1^) was almost 26 times higher than that of the bulk g-C_3_N_4_ structure (1.26 μmol g^−1^). Additionally, the photocurrent stability of this nanoporous 1D morphology over 24 h indicated remarkable photocorrosion resistance. The improved photocatalytic activities were attributed to prolonged carrier lifetime because of its quantum confinement effect, effective separation and transport of charge carriers, and increased number of active sites from interconnected nanopores throughout the microrods. The present 1D nanostructure would be highly suited for photocatalytic water purification as well as water splitting devices. Finally, this facile and room temperature strategy to fabricate the nanostructures is very cost-effective.

Semiconductor photocatalysis is one of the most promising technologies to produce clean hydrogen (H_2_) energy and degrade a wide range of contaminants without toxic byproducts using solar light^1^,^2^,^3^,^4^. Up to now, various types of photocatalysts, such as transition metal oxides (TiO_2_, ZnO, SnO_2_ and WO_3_), chalcogenides (CdS, PbS and CdSe), and metal-organic frameworks, have been exploited effectively to generate H_2_ and degrade various pollutants^5^,^6^,^7^,^8^,^9^,^10^. Unfortunately, their photocatalytic performance is strongly inhibited by rapid charge-carrier recombination, poor light utilization and photocorrosion issues. Moreover, most of the photocatalysts are environmentally hazardous, difficult to recycle, and chemically/thermally unstable, leading to human health concerns and high fabrication costs.

Therefore, a crucial research topic is to develop efficient photocatalysts with broader and high light absorbance, rapid charge transfer, and anti-photocorrosion properties. Polymeric graphitic carbon nitride (g-C_3_N_4_) has been successfully used in water decontamination and splitting because of several key properties, such as it’s being metal free and having a good visible light response (ca. 2.7 eV) and good thermal and chemical stabilities (up to 500 °C; at a pH from acidic to alkaline)^11^. The g-C_3_N_4_ can be synthesized via a single-step polycondensation method with different N-rich precursors including urea, thiourea, melamine, and dicyandiamide, which are readily available at low cost^12^,^13^,^14^,^15^,^16^. Moreover, its unique electronic and optical properties are useful for photocatalytic H_2_ evolution and water purification^17^,^18^,^19^. Despite its many outstanding properties, the photocatalytic performance of g-C_3_N_4_ is severely restricted by a low electronic conductivity, a high rate of photogenerated electron-hole pairs, and a low surface area. Hence, various approaches have been explored and adopted to overcome these problems, such as the fabrication of heterojunctions, sensitization with metal nanoparticles, doping with metallic and non-metallic elements, variation in C and N percentages, delamination of the layered structure, and control of the morphology^20^,^21^,^22^,^23^,^24^,^25^,^26^,^27^,^28^,^29^.

Among these proposed strategies, increasing the number of active sites and speeding up the transport of charge carriers via controlling the morphology, are considered to be the easiest paths to enhance photocatalytic performance. In particular, 1D nanostructures are promising candidates, compared to other bulk materials because of their unique intrinsic properties for light harvesting and photoelectrochemical (PEC) conversion. Many 1D nanostructures exhibit fast and long-distance charge-carrier transport. Moreover, the magnitude of the charge-carrier mobility is far higher than that for nanoparticles. The short diffusion length for the electrons and ions of the electrolyte and the high aspect ratio (length:diameter) increase the optical absorbance because of the more extensive interaction with light and greater surface area compared with the bulk structure^30^,^31^,^32^. These desirable properties of 1D nanostructures make them suited to photocatalysis applications. To date, g-C_3_N_4_ 1D nanostructures such as nano/microfibers, nanorods, microstrings, nanotubes, tubular nanofibers, nanocones, and nanohelices have been successfully synthesized and studied for photocatalytic water splitting and water detoxification^33^,^34^,^35^,^36^,^37^,^38^,^39^. However, the reported morphologies were obtained in multiple steps and required, for example, the use of silicon templates, additional ultrasonication treatments, or plasma sputtering. Those method requires high temperatures (>200 °C) and are expensive because of the high energy demands, which substantially increases the device cost. Additionally, these structures could not achieve the desired photocatalytic efficiency. The aforementioned 1D morphologies were also compact and lacked pores, which restricted the number of active sites for photocatalysis and led to poor device performance. Therefore, it is crucial to develop a low-cost, simple, and low/room temperature process that can overcome aforementioned drawbacks with desirable nanostructures for efficient water splitting.

Herein, we demonstrated a room temperature, template-free approach for the synthesis of a nanoporous g-C_3_N_4_ structure in the form of microrods using simultaneous etching and exfoliation by concentrated acid. The intercalation of acid ions and uneven bonding inside bulk particles resulted in the formation of a 1D morphology with interconnected nanopores. The formed structure showed remarkable photocatalytic activities compared with bulk g-C_3_N_4_ structures. The enhanced activities stemmed from efficient charge-carrier separation and rapid transport, a broader spectral absorbance range, and a large number of active sites for the photocatalysis reactions. We propose the formation mechanism of 1D nanoporous structure from the bulk g-C_3_N_4_ particles. Therefore, the present 1D nanostructures synthesized via top-down strategy may be a revolutionary cost-efficient route to efficient photocatalytic water splitting and degradation of a wide range of water contaminants.

## Results

### FE-SEM and TEM

**Figure 1:**
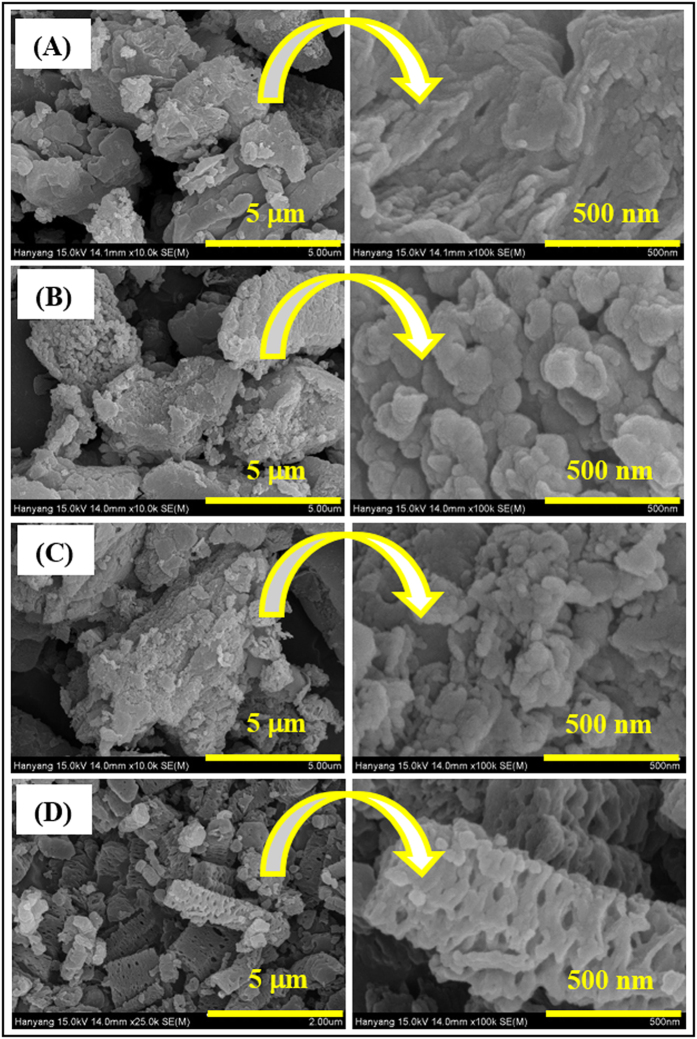
SEM images showing the microstructures of the g-C_3_N_4_ samples before and after the acid treatments. (**A**) BCN, (**B**) HCN, (**C**) NCN, and (**D**) SCN. The images at the right-hand side are at higher magnification.

**Figure 2:**
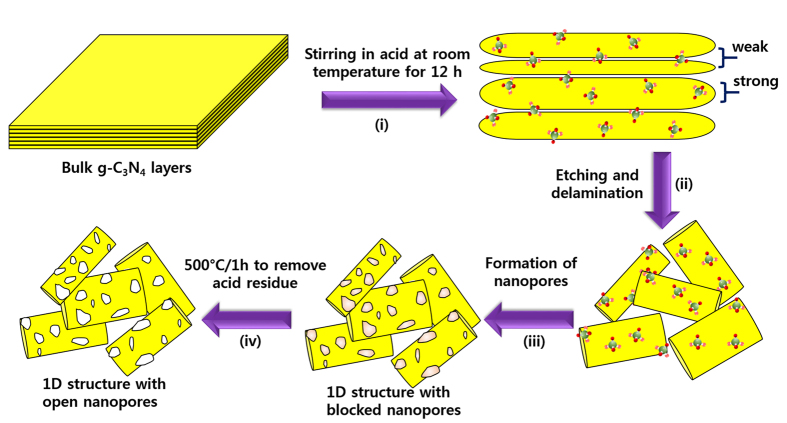
The proposed growth mechanism of the nanoporous 1D structure via a top-down approach at room temperature.

Various approaches have been used to obtain exfoliated graphene and g-C_3_N_4_ layered structures. The intercalation of chemical compounds delaminates stacked graphite into a few or single sheets while preserving its conjugated structure. The bulk graphitic carbon nitride (BCN) powder used in this research was synthesized via the thermal condensation of melamine at 520 °C in air. Figure 1A shows a field-emission scanning electron microscopy (FE-SEM) image of the as-obtained BCN powder. The bulk structure was composed of thick, micron-sized particles, which were unevenly distributed over the entire area of the sample. Most of the particles had multiple stacked layers and rough surfaces. Figure 1B shows the sample morphology after the BCN was treated with concentrated hydrochloric acid (HCN). There was no drastic change in the particle shape and size with the acid treatment, indicating that the hydrochloric acid did not exfoliate the BCN structure. Figure 1C shows FE-SEM images of nitric acid-treated g-C_3_N_4_ powder (NCN). The bulk particles were smaller and had random shapes. Moreover, the presence of agglomerated sheets indicated exfoliation at the small scale. Figure 1D shows that the morphology of sulfuric acid-treated g-C_3_N_4_ (SCN) was completely different from that of the pristine g-C_3_N_4_ structure. Compared with the parent BCN bulk particles, the SCN consisted of micron-sized rods with a large number of pores and rougher and etched surfaces. Almost all of the bulk structure had been converted into rod-like shapes with broad diameter (0.5–1.0 μm) and length (2–4 μm) distributions. Notably, images of broken rods revealed that they were porous. Clearly, sulfuric acid had played a crucial role in converting the bulk particles into porous microrod shapes. Sulfuric acid delaminated the bulk g-C_3_N_4_ structure into individual nanosheets because of its strong oxidizing power^40^. Additionally, because of the large size of the sulfate ion, cations readily intercalated into the bulk structure^41^,^42^,^43^,^44^,^45^. This process created internal stresses and resulted in exfoliation. A similar exfoliation mechanism could have occurred with separation of relatively small particles from the bulk (Fig. 2). This process would result from the intercalation of sulfate ions into the bulk particles, which would induce stresses inside the bulk particles (step i) because BCN has a layered structure with uneven interactions between the layers. Initially, the first few relatively weakly bonded layers would detach from the bulk particles, leading to rod-like particles. Thereafter, most of the bulk particles would be dissected into micron-sized rods (step ii). Simultaneously, sulfate ions would intercalate into the separated particles and form nanopores. This process would continue until most of the weakly bonded particles were separated. Some strongly-bonded particles may not have separated further. Overall, this process converted the bulk form into porous microrods having the g-C_3_N_4_ structure (step iii). Numerous interconnected pores were generated throughout the microrods, forming a nanoporous structure. Simultaneously, residues produced during this process could have blocked some pores (Figure S1), although these blocked pores seem to have been opened during sintering at 500 °C for 1 h in air to form the crystalline g-C_3_N_4_ structure (step iv). We found that sulfuric acid created internally connected pores within the 1D microrods, which improved the number of active sites and charge-carrier transport. In contrast, hydrochloric and nitric acids had minor effects on exfoliation of the BCN structure. Overall, the structures obtained from these acid treatments exhibited irregular and randomly-shaped particles.

**Figure 3:**
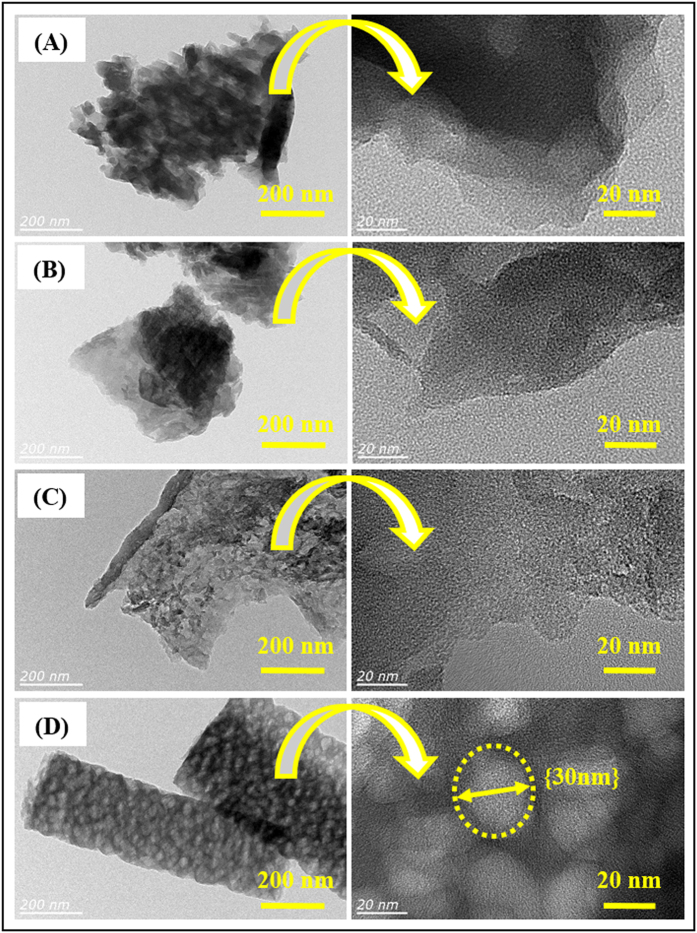
TEM images of (**A**) BCN, (**B**) HCN, (**C**) NCN, and (**D**) SCN. The images at the right-hand side are at higher magnification.

The detailed microstructural features were further characterized using transmission electron microscopy (TEM). The TEM images of BCN in Fig. 3A reveal random shapes of particles that are a few micrometers in size. The darker features in the TEM images are attributed to the stacking of g-C_3_N_4_ layers, resulting in a thick BCN structure. The morphologies of BCN treated with hydrochloric and nitric acids were also studied by TEM (Fig. 3B,C, respectively). The HCN sample consisted of relatively thin structures resembling graphene (Fig. 2B). The lateral size of the sheets was similar to that of the bulk structure; darker areas of the image were assigned to unexfoliated g-C_3_N_4_ layers. The NCN retained the sheet-type structure of the bulk, but had more transparent features and curved edges, possibly because of the small extent of exfoliation of the bulk g-C_3_N_4_ structure that occurred in the presence of nitric acid. Figure 3D clearly shows the presence of nanoporous micron-sized rods about 300 nm in diameter. TEM confirmed that the sulfuric acid treatment converted the random-sized particles of the BCN into the 1D porous SCN structure. Moreover, the formed microrods also exhibited numerous interconnected nanopores, with an average pore diameter of 30 nm (magnified image in Fig. 2D), in agreement with the SEM morphology (see Fig. 1(D)). The porous 1D structure can facilitate mass diffusion via channels for effective photocatalysis.

### XRD

**Figure 4:**
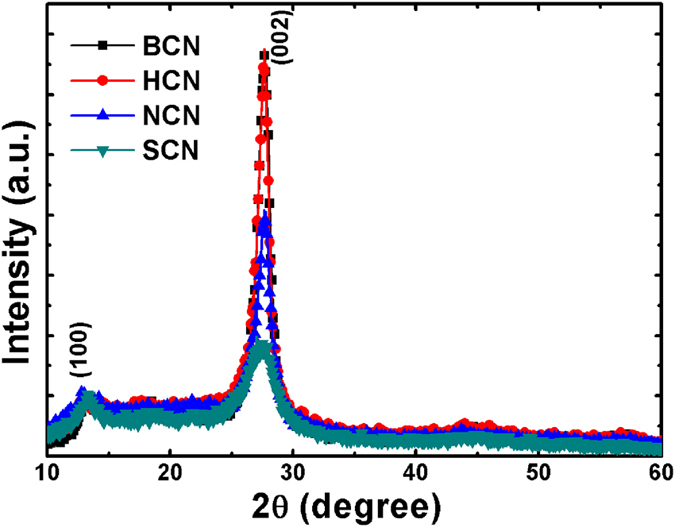
XRD diffraction patterns of BCN, HCN, NCN, and SCN. The inset shows the peak position corresponding to the (002) planes.

X-ray diffraction (XRD) patterns were collected to analyze the phases in the pure bulk and the various acid-treated g-C_3_N_4_ powders. Figure 4 shows that all of the samples exhibited two peaks, indicating the same basic g-C_3_N_4_ atomic structure. Similar results were reported in the literature^46^. The low angle peak at ca. 14.1° originated from in-planar repeated tri-s-triazine units along the (100) plane. The acid-treated powders surprisingly showed similar peak intensities as the bulk structure. This demonstrated that the size of the planar g-C_3_N_4_ feature remained the same even with acid treatment. Another strong peak at 27.68° stemmed from stacked conjugated aromatic systems oriented along the (002) plane. Remarkably, the reduced intensity of the (002) plane peak indicated increased stacking distance between the g-C_3_N_4_ interlayers, indicating disruption of the bulk structure. More importantly, the (002) diffraction peak position shifted from 27.16° from 27.68°, which confirmed the expansion of the interlayer gap^47^. The HCN and NCN powder patterns also displayed small changes in peak intensity compared with BCN, revealing partial separation of the layered g-C_3_N_4_ structure. Additionally, the absence of a peak shift indicated that the HCl and HNO_3_ acids were unable to delaminate the BCN structure. Of the acids evaluated, only H_2_SO_4_ could etch the surface of the bulk g-C_3_N_4_ structure and then convert the surface into a nanoporous 1D rod-like structure. This resulted from the strong oxidation by the H_2_SO_4_ acid. Furthermore, the surface charge of the g-C_3_N_4_ structures was analyzed by measuring the zeta potential; the obtained values are summarized in Table 1. The positive zeta potentials of SCN (36.37 mV), HCN (10.16 mV), and NCN (16.76 mV) indicated that the net charge on their surfaces was negative; the positive values were attributed to protonation. On the other hand, the BCN (–42.67 eV) dispersion showed a negative zeta potential, indicating the presence of net positive surface charge on the bulk g-C_3_N_4_ structure. The great shift in the zeta potential proved that successful etching and exfoliation had occurred during the acid treatment. The delaminated g-C_3_N_4_ structure was thus expected to be more easily dispersed and have better photocatalytic performance because of the increased number of active sites.

### Optical absorbance and FT-IR spectroscopy

**Figure 5:**
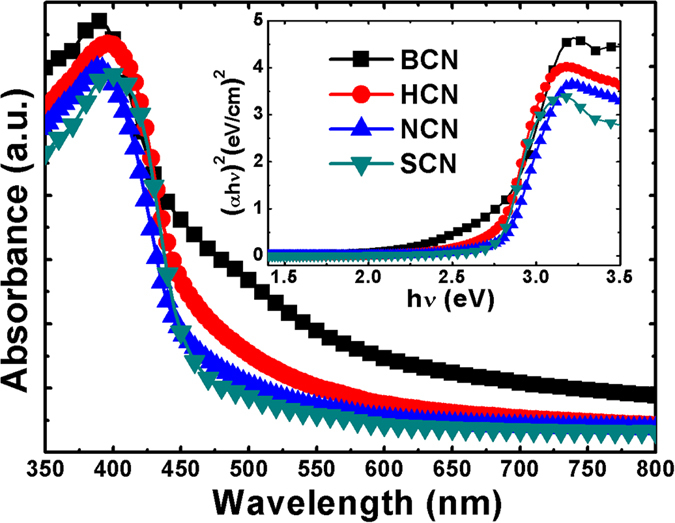
Optical absorbance spectra of BCN, HCN, NCN, and SCN in the range of 350–800 nm. The inset shows the corresponding Kubelka–Munk plots.

The optical properties of the bulk and various acid-treated g-C_3_N_4_ powders were studied by UV-visible absorbance spectroscopy. Figure 5 shows the distinct absorption edges found in the absorbance spectra, indicating a change in bandgap energy from that of the bulk structure. For the acid-treated samples, the absorption edge clearly shifted from a longer to a shorter wavelength (487 to 454 nm). The extension of the band edge could have been caused by the exfoliation and size reduction of the bulk g-C_3_N_4_ structure. The inset of Fig. 5 shows a Kubelka–Munk plot constructed from the optical absorbance data. The absorption edge of the acid-treated g-C_3_N_4_ powders was significantly blue-shifted from that of the bulk structure. The highest bandgap energy of 2.72 eV was observed for the SCN as compared to that of the bulk structure (2.54 eV). The sample treated with HCl acid had a bandgap energy of 2.63 eV, and it was 2.67 eV for the nitric acid-treated one. This was attributed to a strong quantum confinement effect resulting from changes in the positions of the conduction and valence bands^48^. The shift in the band position is beneficial to increase reduction and oxidation potentials. A porous 1D structure also improves charge transfer process that could increase the photocatalytic performance.

**Figure 6:**
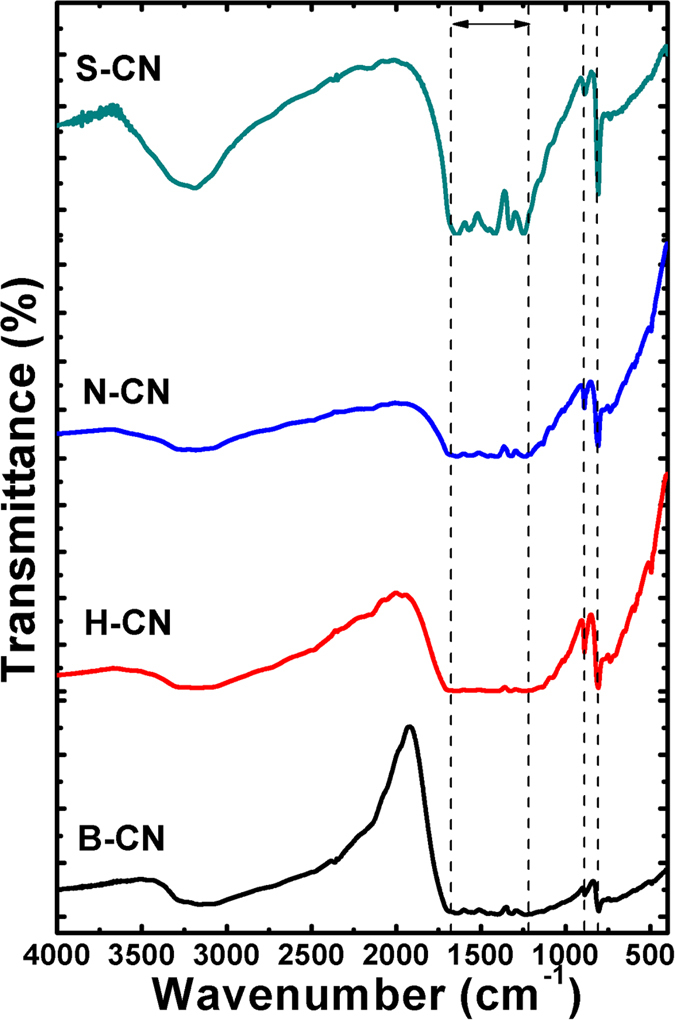
FT-IR spectra of pristine g-C3N4 and treated with the different acids.

The chemical composition and structural changes were investigated by FT-IR spectroscopy (Fig. 6). The characteristic peaks for the acid-treated samples were similar to those of the bulk structure, indicating that g-C_3_N_4_ had the same chemical composition even after exfoliation and conversion of the morphology. The many small peaks observed in the range of 1200–1650 cm^−1^ correspond to stretching vibrations of C=N, C–NC–C or C–N–C bonds of C–N heterocycles^49^,^50^. The presence of these bands confirmed that the basic surface functional units were retained after the acid and thermal treatments. The sharp band at 810 cm^−1^ corresponds to the out-of-plane breathing modes of the heterocyclic triazine ring^48^. The small, sharp band at ca. 880 cm^−1^ was assigned to the stretching mode of the N–O group^51^. Additionally, the broad band at 3500 cm^−1^ was assigned to the stretching mode of the hydroxyl group. Curiously, this band was strongest for the SCN sample, which may have resulted from slight oxidation of the g-C_3_N_4_ by the strongly oxidizing sulfuric acid^52^. However, the presence of most of the characteristic bands in the acid-treated samples indicated that layered g-C_3_N_4_ triazine bonds persisted and were perhaps only slight oxidized.

### Brunauer-Emmett-Teller (BET) surface area and XPS analysis

To demonstrate the increased active sites after acid treatment, we measured BET surface area of all g-C_3_N_4_ samples. Figure S2(A to D) show the nitrogen adsorption-desorption isotherms with hysteresis of type IV behavior indicating the mesoporous structure of g-C_3_N_4_ with number of active sites. The specific surface area of HCN (15.95 m^2^ g^−1^), NCN (58.82 m^2^ g^−1^), and SCN (32.05 m^2^ g^−1^) samples are found to be considerably higher than that of BCN (7.46 m^2^ g^−1^). This confirms that the acid treatment encourages exfoliation and hence number of active sites for adsorption of contaminants. The enhanced active sites are beneficial to improve photodegradation and water splitting performances.

The chemical states of the bulk and acid-treated g-C_3_N_4_ structures were analyzed using X-ray photoelectron spectroscopy (XPS). Survey spectra revealed two major peaks corresponding to carbon (C) and nitrogen (N) elements (Figure S3A). An additional oxygen (O) peak indicated protonation of the g-C_3_N_4_ by acid. The intensity of the O peak was significantly higher for SCN than for the other bulk and acid-treated samples, indicating oxidation of the g-C_3_N_4_ by the sulfuric acid. Four distinctive peaks were observed in the deconvoluted C 1s feature (Figure S3B). The two major peaks at 287.93 (C1) and 284.60 eV (C3) were assigned to hybridized carbon in the triazine ring and carbon nitride with low graphitic content^53^. The peak at 285.94 eV (C2) of relatively low intensity was attributed to the aromatic structure of sp^2^ carbon atoms bonded with nitrogen^54^. The additional peak at 283.16 eV (C4) was attributed to carbon contamination. The deconvoluted N 1s band showed two peaks at 400.28 (N1) and 398.25 eV (N2) that were assigned to the pyrollic and pyridinic nitrogen centers in triazine rings (Figure S3C). The shift of the N2 peak toward lower binding energy may have resulted from oxidation of the g-C_3_N_4_ structure^55^,^56^. The C/N molar ratios calculated from the XPS analyses are summarized in Table TS1. That for SCN (1.97) was almost double those of BCN (0.93), HCN (0.99) and NCN (1.01), which confirmed the loss of nitrogen during the acid treatment and the creation of vacancies. Hence, the XPS study clearly demonstrated that the oxidation process greatly reduced the amount of nitrogen, which could assist in increasing photocatalytic activities.

### Photodegradation of MB dye

**Figure 7:**
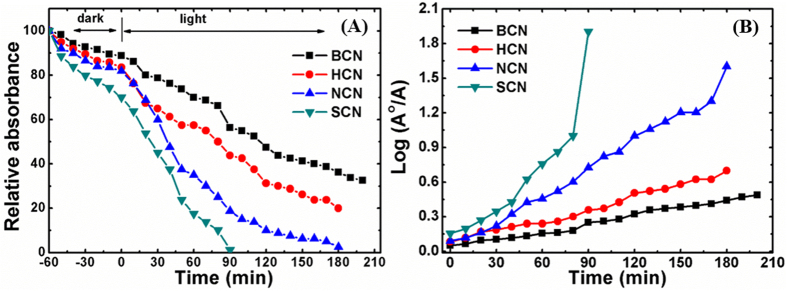
(**A**) Relative MB concentrations during photocatalysis for the pure and acid-treated g-C_3_N_4_ microstructures. The absorbance of MB without photocatalysis is also shown for comparison. (**B**) Photodegradation of MB as a function of time with and without catalysts.

The acid-treated g-C_3_N_4_ structures were expected to show better photocatalytic performance compared with the bulk because of their broader absorption range and larger number of active sites generated from exfoliation. The nanoporous 1D morphology of the SCN sample was particularly favorable for rapid charge-carrier transport, which was expected to provide excellent photocatalytic performance. The photocatalytic performance of all of the g-C_3_N_4_ samples was evaluated by degradation of methylene blue (MB) dye as a simulated pollutant under visible light irradiation (Fig. 7A). Additionally, self-adsorption of dye molecules was verified by treatment in the dark for 60 min. The increased adsorption by all of the samples in the dark indicated a slight removal of MB molecules. Furthermore, the photocatalytic performance of the acid-treated g-C_3_N_4_ powders indicated a more rapid degradation rate than for the bulk sample. Notably, the SCN sample degraded almost 100% of the dye within 90 min of visible-light irradiation, whereas the HCN and NCN samples degraded about 56 and 80% of the dye, respectively. However, the BCN sample only degraded about 44% of the dye. Furthermore, we compared photocatalytic activity of sintered BCN sample to confirm the role of temperature (degradation =54%, Figure S4A). The photodegradation performance, being quite similar with that of BCN, revealed that there is no sintering effect on photodegradation of MB. Finally, these results clearly indicated that the SCN more readily degraded MB than HCN, NCN, and BCN did.

The photodegradation reaction kinetics of all of the g-C_3_N_4_ structures were quantified by fitting the decay of the peak at 663 nm to the function ln(*A*_0_/*A*) = *kt*, where *k* is the apparent kinetic rate constant, *A*_0_ is the initial absorbance of the MB solution, and *A* is the MB absorbance at time *t*. The logarithmic plots (Fig. 7B) were straight lines, indicating pseudo-first-order kinetics. Table 2 lists the calculated rate constants for all samples. The SCN sample had the highest kinetic rate constant (15.61 min^−1^), which was substantially higher than that for the BCN (2.29 min^−1^) and the other acid-treated photocatalysts, i.e., HCN (3.35 min^−1^) and NCN (7. 95 min^−1^). It was apparent that the acid treatment of the bulk g-C_3_N_4_ structure remarkably reduced recombination losses and improved the photocatalytic performance.

### Photocatalytic H_2_ evolution

**Figure 8:**
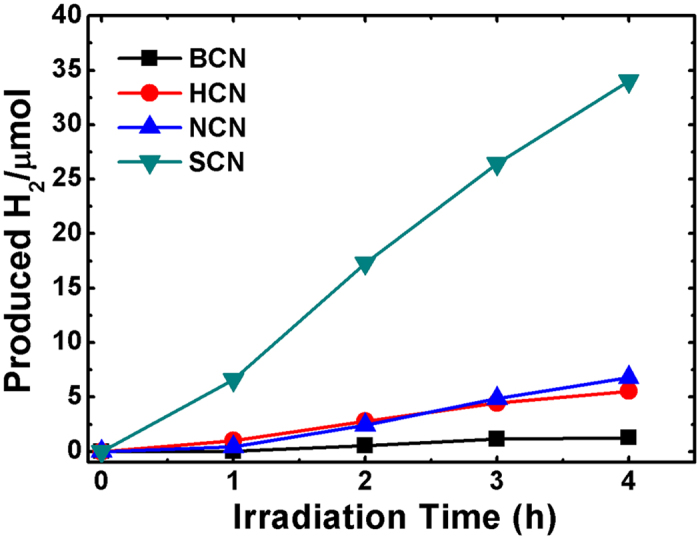
Photocatalytic H_2_ evolution of BCN, HCN, NCN, and SCN samples.

The photocatalytic H_2_ evolution activities of the g-C_3_N_4_ structures were conducted by irradiating 50 mL of a 10 vol% triethanolamine (TEOA) solution with visible light. The H_2_ evolution of the HCN, NCN and sintered-BCN samples was measured under the same experimental conditions for comparison. Figure 8 presents the H_2_ evolution curves of the bulk and acid-treated g-C_3_N_4_ samples. The SCN had a significantly enhanced H_2_ evolution rate (34.00 μmol g^−1^) compared with that of BCN (1.26 μmol g^−1^) and the other acid-treated samples. The H_2_ evolution rates of HCN (5.54 μmol g^−1^) and NCN (6.80 μmol g^−1^) were higher than that of the bulk sample, but far lower than that for the SCN material. The sintered-BCN sample, also exhibited poor performance, confirming that sintering of g-C_3_N_4_ has no effect on exfoliation and photocatalytic performances (1.49 μmol g^−1^, Figure S4B). In addition, the present H_2_ evolution rate is comparable/higher than that of reported in the literature for pristine g-C_3_N_4_ structures (Table TS2). A similar trend was observed with the photodegradation of MB dye under visible-light irradiation. Hence, the porous SCN structure with its unique interconnected pore structure provided a large contact area and a large number of active sites, which improved the charge-carrier transport, reduced recombination losses, and thereby improved the H_2_ evolution rate. Clearly, the nanoporous 1D structure of g-C_3_N_4_ is a promising photocatalyst for water splitting applications.

## Discussion

### Charge-carrier separation and transport

**Figure 9:**
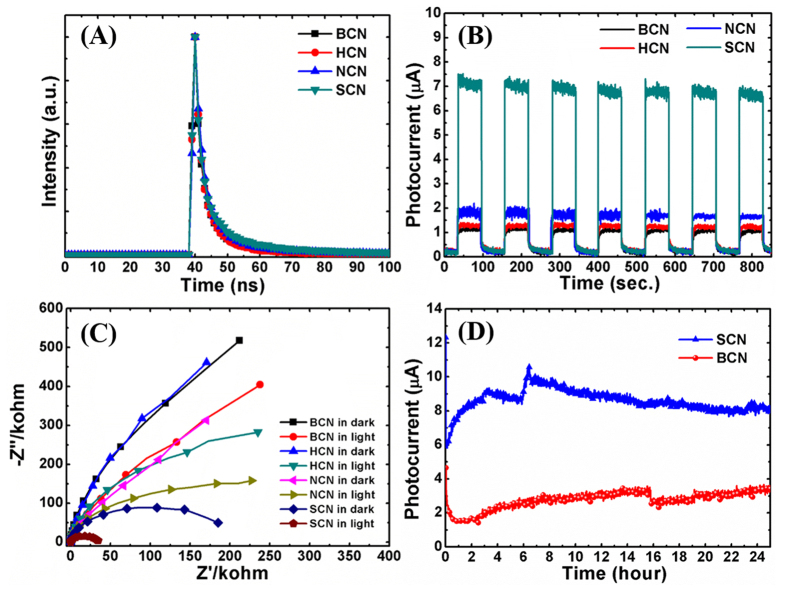
(**A**) Time-resolved photoluminescence (TRPL) spectra of (**A**) BCN, (**B**) HCN, (**C**) NCN, and (**D**) SCN. (**B**) Photoelectrochemical measurements showing the transient response of BCN, HCN, NCN, and SCN. The measurements were taken under visible-light irradiation in 0.5 M Na2SO4 electrolyte. (**C**) EIS spectra of BCN, HCN, NCN, and SCN measured in the dark and under visible-light irradiation in 0.5 M Na_2_SO_4_ electrolyte. (**D**) Photocurrent stability of BCN and SCN under visible-light irradiation for 24 h.

Photocatalytic performance mainly depends on the number of active sites, the extent of light absorption, and the effective generation/separation/transport of charge carriers. The great number of active sites and direct transport paths for rapid charge carriers within 1D nanostructures could enhance charge-carrier separation. In the present work, we generated a porous 1D nanostructure of g-C_3_N_4_ via a facile chemical approach at room temperature. The photocatalytic degradation of MB and water splitting results indicated that the 1D structure of g-C_3_N_4_ more effectively degraded MB and generated H_2_ more rapidly compared with the bulk and sheet-type samples. The superior photocatalytic performance was attributed to efficient separation and migration of photogenerated charge carriers. Time-resolved photoluminescence (TRPL) emission spectra were recorded to study the dynamics of the charge carriers in the exfoliated and bulk g-C_3_N_4_ structures. Figure 9A shows the TRPL spectra of all of the photocatalysts measured at room temperature. The TRPL data were fitted to a double-exponential function, and the average carrier lifetime was calculated from Equation (1)^57^ as follows:


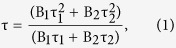


where, τ_1_, τ_2_, B_1_, and B_2_ are constants corresponding to radiative and nonradiative deactivation processes. Table 3 lists the fitted parameters of the TRPL decay spectra of all samples. The SCN sample had the highest exciton lifetime, 9.75 ns, which was almost double that of the BCN sample (5.43 ns). Moreover, the carrier lifetimes of HCN (6.88 ns) and NCN (7.53 ns) were also higher than that of the bulk sample. The prolonged carrier lifetime for the porous and 1D microrod-like structure of g-C_3_N_4_ enhanced the photocatalytic efficiency. Additionally, the longer charge-carrier lifetime enabled its involvement in the photocatalytic reaction processes. The TRPL results confirmed that the nanoporous 1D structure would be better than the bulk and other exfoliated structures for water purification applications.

### Transient photoresponse

The transient photocurrent response of the g-C_3_N_4_ structure was measured to understand the efficient separation and charge-carrier transport. Samples were subjected to several cycles of intermittent irradiation with visible light. Figure 9B depicts the transient photocurrent as a function of time for BCN, HCN, NCN, and SCN working electrodes at a bias potential of 1 V. The photocurrent increased rapidly as soon as the light was turned on, and then returned to its original position quickly once the light was turned off. This transient phenomenon could be repeated over several cycles. The increased photocurrent indicated photoactivity of the synthesized g-C_3_N_4_ structures. Notably, the photocurrent of the SCN (7.09 μA) was considerably higher than those of the BCN (1.12 μA), HCN (1.33 μA), and NCN (1.87 μA) samples. These results indicated enhancement in charge-carrier transport with fewer recombination losses. The highest photocurrent was observed for the SCN sample and was attributed to more rapid transport of charge carriers in the 1D nanostructure over a broader light absorption range. These results also supported the enhanced photodegradation of MB and improved H_2_ evolution rate.

### EIS measurements in the dark and in light

The interfacial charge transfer rate is crucial for enhanced photocatalytic performance. Electrochemical impedance spectroscopy (EIS) analyses in the dark and under illumination provide direct evidence concerning charge separation and its contribution to the degradation of contaminants and H_2_ evolution. Therefore, we conducted EIS analysis to further investigate the separation and transfer efficiency of charge carriers. Figure 9C shows the EIS Nyquist plots of all of the g-C_3_N_4_ photoelectrodes in the presence and absence of visible-light irradiation. The results showed that the interfacial charge transfer resistance for the acid-treated samples was lower than that of the bulk. Notably, the 1D structure synthesized using sulfuric acid had the lowest interfacial charge transfer resistance, which resulted in remarkable charge separation and transport. Additionally, the significant decrease in the impedance for the acid-treated samples indicated the generation of a large number of photoelectrons during irradiation. This again indicated that the acid-treated samples had more effective separation of charge carriers and lower interfacial charge transfer resistance^58^. Thus, the EIS results indicated less recombination with the acid-treated g-C_3_N_4_ than for the bulk, i.e., improved photocatalytic activities.

### PEC stability test

The durability of photocatalysts is important for practical device applications. Usually, photocorrosion of a catalyst degrades its efficiency and reduces its long-term stability^59^,^60^. Figure 9D compares the photocurrent stability over 24 h for the SCN and BCN samples under the same experimental conditions. The steady generation of photocurrent over a prolonged irradiation time demonstrated the great stability of the nanoporous 1D structure of g-C_3_N_4_. To our best knowledge, this is the first report of the steady generation of photocurrent from g-C_3_N_4_ structure over such a long irradiation time. Hence, the present 1D porous g-C_3_N_4_ structure not only improved charge carrier transport but also was stable toward photocorrosion. Therefore, the synthesized g-C_3_N_4_ nanostructure holds great promise for rapid degradation of organic pollutants and for efficient water splitting applications.

### Photocatalysis mechanism

The above analyses indicated that the 1D nanostructure of g-C_3_N_4_ displayed remarkable photocatalytic performance under visible-light irradiation owing to effective separation and transport of charge carriers. The presence of nanopores on the microrods increased the number of active sites for dye adsorption and water splitting. Additionally, TRPL and EIS analyses confirmed longer carrier lifetimes and lower charge transfer resistances for the 1D nanostructures compared with the bulk. The bulk structure had poor performance due to the high rate of recombination losses. A photocatalysis mechanism was developed based on these findings. Overall charge generation and transport processes are given in Equations (2)–(5)^61^,^62^,^63^,^64^:


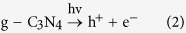



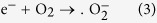



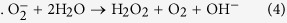



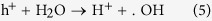


**Figure 10:**
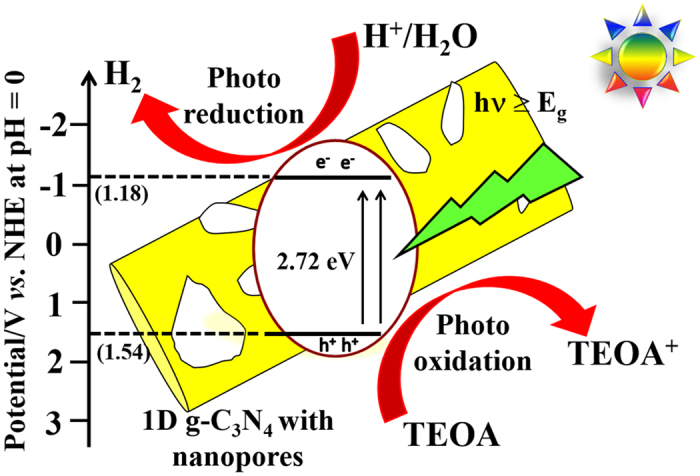
Photogeneration and electron transport mechanism under visible-light irradiation for photocatalytic water splitting with the nanoporous 1D g-C_3_N_4_.

The acid treatment inducing the exfoliation of bulk into few layered structure, resulted in a number of active sites and significantly enhanced the charge carrier’s mobility. Simultaneously, the presence of quantum confinement effect could have boosted the redox capacity of charge carriers. Figure 10 shows the electron transfer and water splitting process by a 1D nanoporous g-C_3_N_4_ structure under visible-light irradiation. Initially, visible light is absorbed to generate electron–hole pairs (Equation (2)). The photogenerated electrons then react with oxygen to form transient superoxide radicals and superoxide molecules (Equations (3) and (4)). Simultaneously, an oxidation reaction generates ·OH radicals, which would neutralize MB molecules (Equation (5)). This reaction mechanism accounts for the capability of the nanoporous 1D structure of g-C_3_N_4_ to rapidly detoxify and split water under visible-light irradiation. This structure also significantly reduces the recombination losses and thereby boosts the photocatalytic performance.

Summarizing, we have produced a 1D nanostructure of g-C_3_N_4_ with a large number of interconnected pores via a facile, template-free chemical process at room temperature. Nanopores were generated throughout the 1D microrods via etching and protonation by acid treatment. These pores induced the number of active sites for adsorption of contaminants. Simultaneously, this unique nanoporous 1D structure leads to rapid electron transfer and separation. These improved properties led to remarkable H_2_ generation and MB photodegradation compared with bulk g-C_3_N_4_ under visible-light irradiation. Consequently, record-high photocorrosion stability (>24 h) was achieved because of much lower radiation recombination loss and increased carrier lifetime. The research reported herein offers an exceptional new type of g-C_3_N_4_ nanostructure that could be readily manufactured at a commercial production scale and applied to water splitting and purification applications. Moreover, this work could open up new opportunities in the optoelectronic and electrocatalytic fields.

## Methods

### Chemicals

All chemicals were purchased from Junsei Chemical Co., Ltd. (melamine) and Dae-Jung Co., Ltd. (HCl, HNO_3_, and H_2_SO_4_) and were used without further purification.

### Preparation of bulk graphitic carbon nitride (BCN)

BCN powder was prepared according to a procedure described in the literature^11^. Briefly, 10 g of melamine powder was placed in an alumina crucible and covered. The crucible was positioned at the center of a muffle furnace and heated at 520 °C for 4 h at the heating rate of 10 °C/min in air. The furnace was cooled to room temperature following completion of the reaction. The formed yellow-colored powder was collected and finely ground. The fine particulate powder was then used in the described experiments and analyses.

### Preparation of acid-treated BCN

The bulk structure of g-C_3_N_4_ was degraded into nanoporous microrods via chemical means at room temperature as follows. The BCN powder (2 g) was added to 100 mL of concentrated H_2_SO_4_ in a 250 mL conical flask under constant magnetic stirring to form a pale-yellow-colored solution. Stirring was continued for 12 h at room temperature. Thereafter, 100 mL of distilled water (DW) was added gradually to the flask. This experiment was conducted in the fume-hood to avoid toxicity problem because of vapors produced during water addition. The color of the solution changed from yellow to colorless within a few seconds, and then turned to white after a few minutes. This change in color corresponded to exfoliation and deformation caused by the acid treatment. The heat generated during the acid addition was controlled by keeping the flask in an ice bath; the DW was added to the ice-cold solution. The resulting white-colored solution was magnetically stirred for about 12 h at room temperature, after which, it was centrifuged at 5000 rpm for 5 min and washed several times with DW until its pH became neutral. The white-colored dense suspension was collected and dried in a vacuum oven at 60 °C to evaporate the water. Finally, the obtained white powder was sintered at 500 °C (at a heating rate of 2 °C/min) for 2 h in a muffle furnace in air to remove residues and to form pure g-C_3_N_4_. For comparison, the BCN powder was also separately treated with concentrated HCl and HNO_3_ acids under the same experimental conditions. The obtained powders from the different acid treatments were labeled as HCN (HCl-g-C_3_N_4_), NCN (HNO_3_-g-C_3_N_4_), and SCN (H_2_SO_4_-g-C_3_N_4_).

### Characterization

Scanning electron microscopy (SEM; Hitachi S4800) was used to study the conversion of the bulk type of g-C_3_N_4_ into the porous microrod and sheet-like structures formed via the acid treatments. Transmission electron microscopy (TEM; JEOL 2100) images of the microstructures were examined at 200 kV. X-ray diffraction (XRD) patterns for the powder were obtained with a Rigaku D/MAX-2500/PC diffractometer with Cu Kα radiation (λ = 0.15418 nm) at 40 kV and 100 mA at room temperature. The zeta potential was measured with a zeta potential analyzer (ELSZ–1000; Photal Otsuka Electronics). Prior to zeta potential analysis, a photocatalyst powder (0.1 mg mL^−1^) was dispersed in water without adjusting the pH by sonicating for 1 h. The UV-visible optical absorption spectra were measured with a V–600 spectrophotometer using a dry-pressed BaSO_4_ disk as reference. Room-temperature photoluminescence (PL) spectra were measured at an excitation wavelength of 325 nm using a He–Cd laser attached to a fluorescence spectrophotometer (Dong Woo Optron). Fourier transform infrared (FT-IR) spectra were recorded at a resolution of 1 cm^−1^ with KBr pellets at ambient temperature. The specific surface area (*S*_BET_) was determined using nitrogen adsorption–desorption isotherms with a Quantochrome machine cooled to liquid nitrogen temperatures (AS1). X-ray photoelectron spectroscopy (XPS) measurements were performed on a Sigma Probe instrument (Thermo Fisher Scientific) to examine the bonding configurations of the carbon and nitrogen atoms. TRPL spectra were recorded with an F7000 fluorescence spectrometer equipped with a femtosecond pulsed laser (excitation wavelength = 325 nm) to study the photoexcited charge-carrier dynamics. The zeta potential of the g-C_3_N_4_ suspension at neutral pH was measured by a ZetaProbe.

### Photocatalytic activity

The photocatalytic behavior of the g-C_3_N_4_ powder was evaluated by photodegradation of MB dye under visible-light irradiation. A similar experimental setup to that reported previously was used^44^. Briefly, photocatalyst powder (50 mg) was dispersed in an MB solution (250 mL, 3.2 mg L^−1^) in a batch-type glass reactor. The reaction solution was stirred for 60 min. in the dark to reach adsorption–desorption equilibrium, and then the lamp was turned on for visible-light irradiation. All measurements were conducted at room temperature with constant stirring (200 rpm). During irradiation, aliquots (3 mL) were withdrawn at 10-min intervals, and their UV-visible absorbances were measured. The MB degradation was monitored by observing the decrease in the optical density at 663 nm.

### Photocatalytic water splitting

The photocatalytic hydrogen (H_2_) evolution experiments were performed using a four-neck quartz reactor at ambient temperature and pressure. Initially, photocatalyst powder (25 mg) was suspended in aqueous triethanolamine solution (50 mL,10 vol%) with constant stirring. Then, 0.5 wt% of Pt co-catalyst (Chloroplatinic acid hexahydrate) was loaded by adding 30 μL of an aqueous solution to the above suspension. Irradiation with a UV lamp (300 W, 365 nm) for 15 min formed Pt nanoparticles. The suspension was purged with nitrogen gas for 30 min to remove residual oxygen before each experiment. Then, the suspension was poured into the reactor and sealed with a rubber septum. The sealed reactor was purged with argon gas (5 mL/min) for 30 min to ensure anaerobic conditions. A 300 W xenon arc lamp (Newport, model 69911) coupled with a 400-nm UV cut-off filter was used as a visible-light source; samples were irradiated for 4 h. The measured incident radiant power was 73 mW cm^−2^ at the position of the reactor. Evolution of H_2_ gas was analyzed by chromatography (YL Instruments, model 6500GC, equipped with a thermal conductivity detector (TCD)). Hydrogen gas (150 μL) was collected from the reactor with a syringe and injected into the gas chromatography column at 1-h intervals.

### Electrochemical measurements

The electrochemical analysis was performed using a standard three-electrode cell and computer- controlled potentiostat (Princeton Applied Research, model VersaSTAT 4) with a graphite counter electrode and Ag/AgCl (saturated KCl) reference electrode. The working electrodes were prepared as follows: 10 mg of g-C_3_N_4_ powder was added to 10 mL of Liquion solution, and the mixture was ultrasonicated for 1 h. Next, the solution was stirred overnight to improve its distribution and to stabilize the viscosity. Then, the white-colored suspension was spin-coated (100 rpm for 60 s) on a fluorine-doped tin oxide (FTO) glass substrate at room temperature. Finally, the FTO films spin-coated with the catalysts (deposit area = 1 cm^2^) were placed in an oven at 60 °C for 2 h to obtain a uniform distribution of the catalyst on the substrate. The electrochemical measurements were conducted in 0.5 Na_2_SO_4_ redox electrolyte solution. The photocurrent was recorded under pulsed visible-light irradiation with the 300 W Xe lamp. Electrochemical impedance spectroscopy (EIS) analysis using the above-mentioned three-electrode experimental setup was conducted over the frequency range of 0.01–10^5^ Hz at an AC amplitude of 5 mV. The EIS data were collected under light and dark conditions in the 0.5 M Na_2_SO_4_ electrolyte.

## Additional Information

**How to cite this article**: Pawar, R. C. *et al.* Room-temperature synthesis of nanoporous 1D microrods of graphitic carbon nitride (g-C_3_N_4_) with highly enhanced photocatalytic activity and stability. *Sci. Rep.* **6**, 31147; doi: 10.1038/srep31147 (2016).

## Supplementary Material

Supplementary Information

## Figures and Tables

**Table 1:** Zeta potential values obtained for BCN, HCN, NCN, and SCN samples.

Sample name	Zeta potential (mV)
BCN	−42.67
HCN	10.16
NCN	16.76
SCN	36.37

**Table 2:** Summary of the kinetic rate constants obtained from logarithmic plots of the MB degradation.

Sample	Kinetic rate constant (min^−1^) × 10^−3^
BCN	2.29
HCN	3.35
NCN	7.95
SCN	15.61

**Table 3:** Summary of the data obtained from time-resolved photoluminescence (TRPL) analyses.

Sample	**τ**_1_	**τ**_2_	B1	B2	Average carrier life time **τ** (ns)	**χ**^2^
BCN	2.22	9.10	7387.84	1578.42	5.43	0.98
HCN	2.67	12.92	6914.12	998.03	6.88	1.13
NCN	2.86	13.47	7348.60	1226.78	7.53	1.12
SCN	2.84	15.87	6381.52	1286.49	9.75	1.24

The English in this document has been checked by at least two professional editors, both native speakers of English. For a certificate, please see: http://www.textcheck.com/certificate/2oXB36.
