# Supplementary material for: Room-temperature synthesis of nanoporous 1D microrods of graphitic carbon nitride (g-C3N4) with highly enhanced photocatalytic activity and stability
Source: Sci Rep. 2016 Aug 8;6:31147. doi: 10.1038/srep31147 (PMC4976354; doi:10.1038/srep31147)
Supplement: Supplementary Information [file srep31147-s1.pdf]

Supporting information

Room-temperature synthesis of nanoporous 1D microrods of graphitic carbon nitride (g-C_3N_4) with highly enhanced photocatalytic activity and stability

Rajendra C. Pawar[1], Suhee Kang[1], Jung Hyun Park[2], Jong-ho Kim[2], Sunghoon Ahn[3] and Caroline S. Lee[1]*

[1]Department of Materials Engineering, [2]Department of Chemical Engineering, Hanyang University, South Korea 426-791.

[3]School of Mechanical & Aerospace Engineering, Seoul National University, South Korea151-742.

Supporting Figure captions

Figure S1. Blocked nanopores of the SCN sample before sintering in air.

Figure S2 Nitrogen adsorption-desorption isotherms of **(A)** BCN, **(B)** HCN, **(C)** NCN and **(D)** SCN.

Figure S3 (A) Survey XPS spectra of BCN, HCN, NCN, and SCN. **(B)** High-resolution spectra of the C 1s regions of BCN, HCN, NCN, and SCN. **(C)** High-resolution spectra of the N 1s regions of BCN, HCN, NCN, and SCN.

Figure S4 (A) Comparison of photocatalytic degradation of bulk and sintered g-C_3N_4 samples and **(B)** Photocatalytic H_2 evolution of bulk and sintered g-C_3N_4 samples.

Table TS1. Summary of the total carbon and nitrogen atomic weight percentages in the BCN, HCN, NCN, and SCN samples.

Table TS2. Comparison of H_2 evolution reported in the literature and present work for pristine g-C_3N_4 structures.

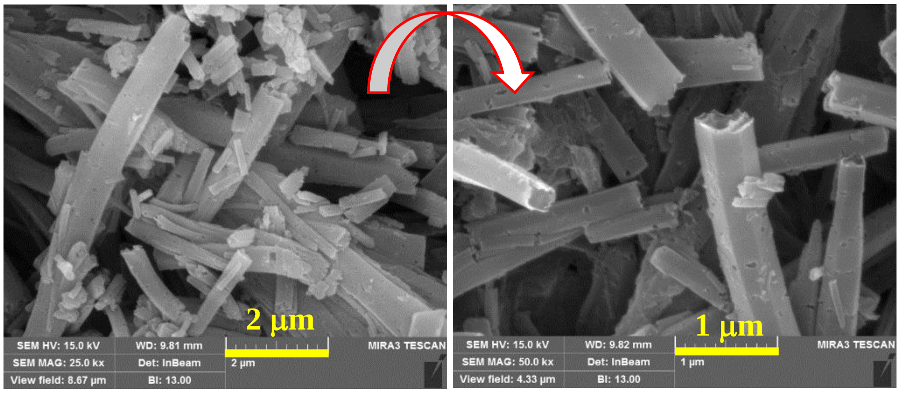

2 µm
SEM HV: 15.0 kV
WD: 9.81 mm
SEM MAG: 25.0 kx
Det: InBeam
View field: 8.67 µm
BI: 13.00
2 µm
MIRA3 TESCAN
1 µm
SEM HV: 15.0 kV
WD: 9.82 mm
SEM MAG: 50.0 kx
Det: InBeam
View field: 4.33 µm
BI: 13.00
1 µm
MIRA3 TESCAN

Figure S1

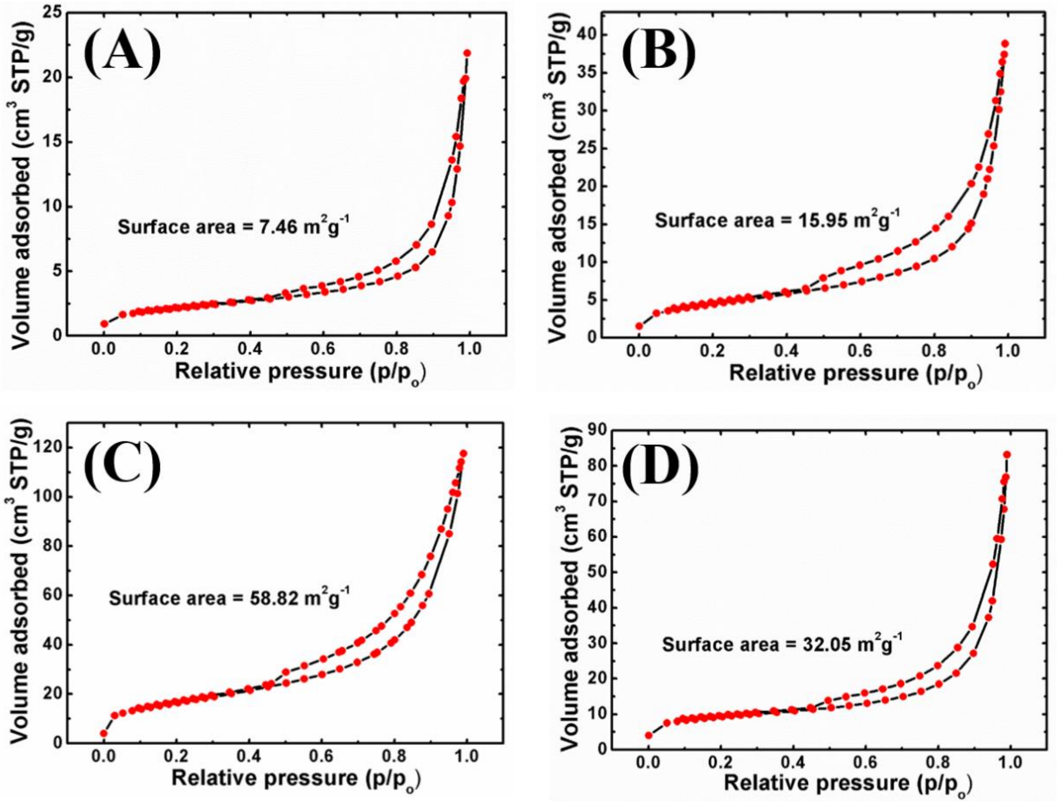

Figure S2 (A to D)

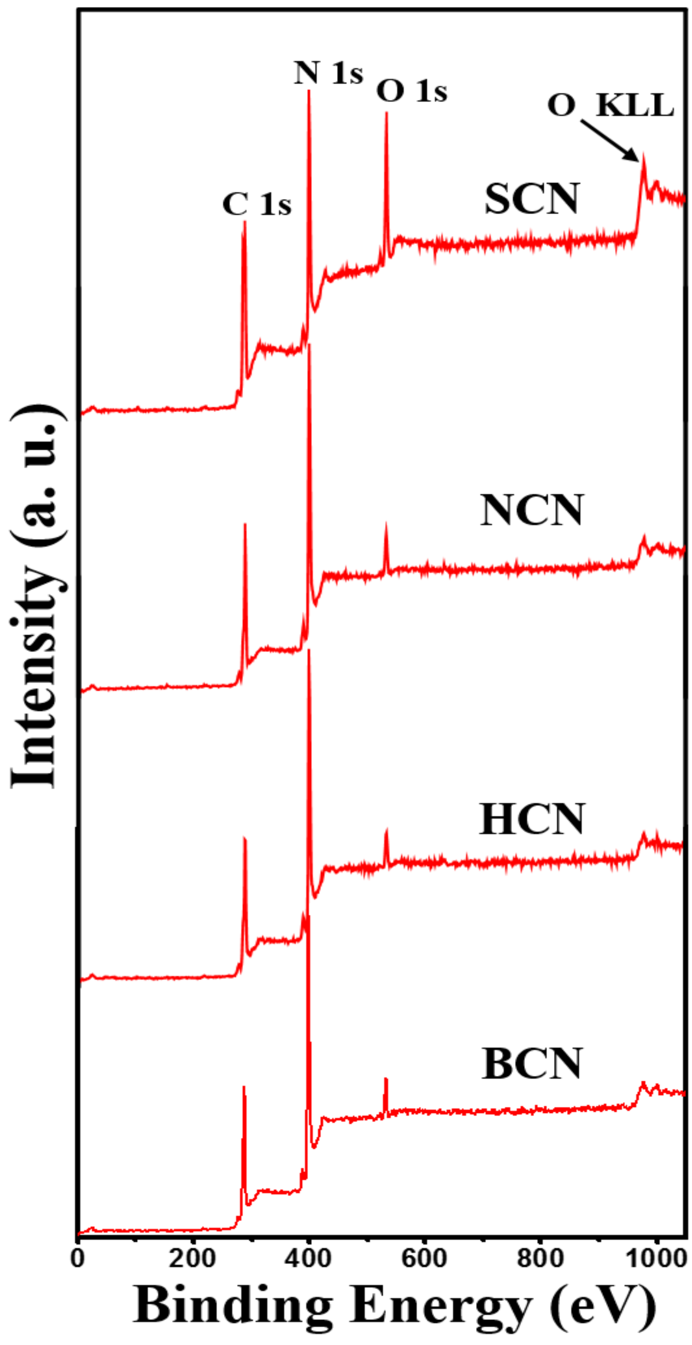

N 1s
O 1s
O KLL
C 1s
SCN
NCN
HCN
BCN
Intensity (a. u.)
0
200
400
600
800
1000
Binding Energy (eV)

Figure S3A

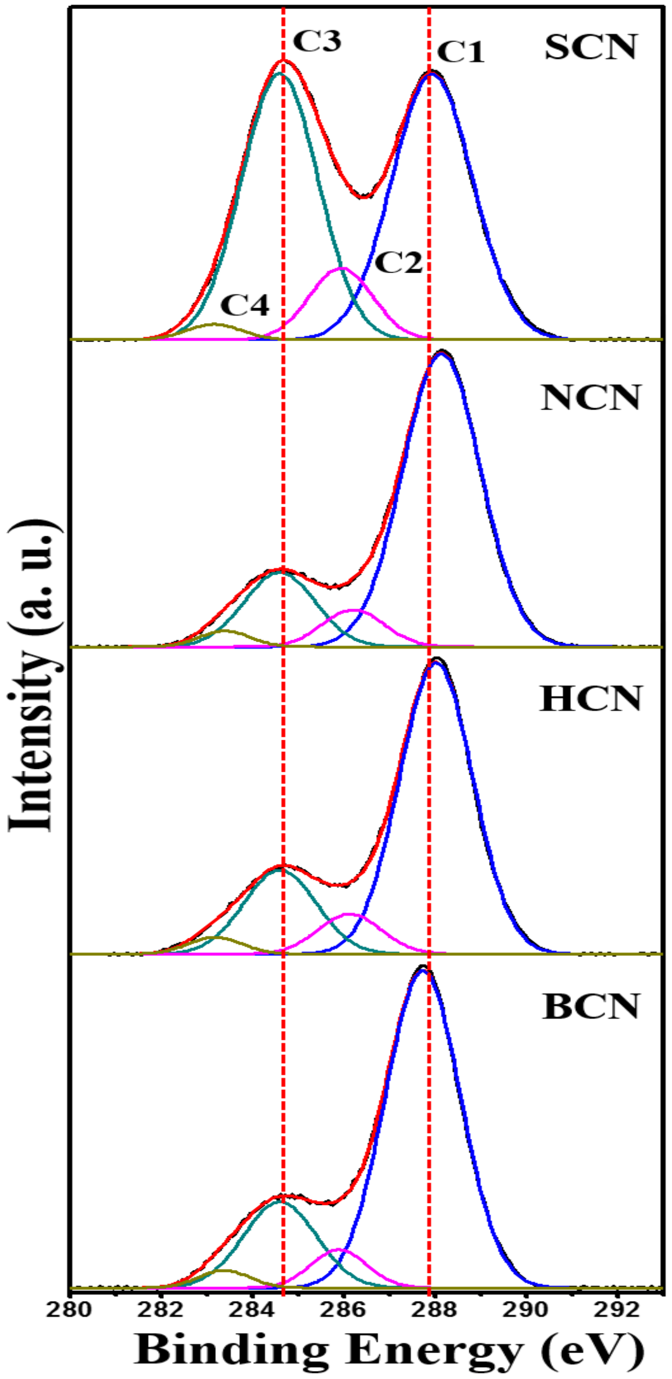

C3
C1
SCN
C2
C4
NCN
HCN
BCN
Intensity (a. u.)
280
282
284
286
288
290
292
Binding Energy (eV)

Figure S3B

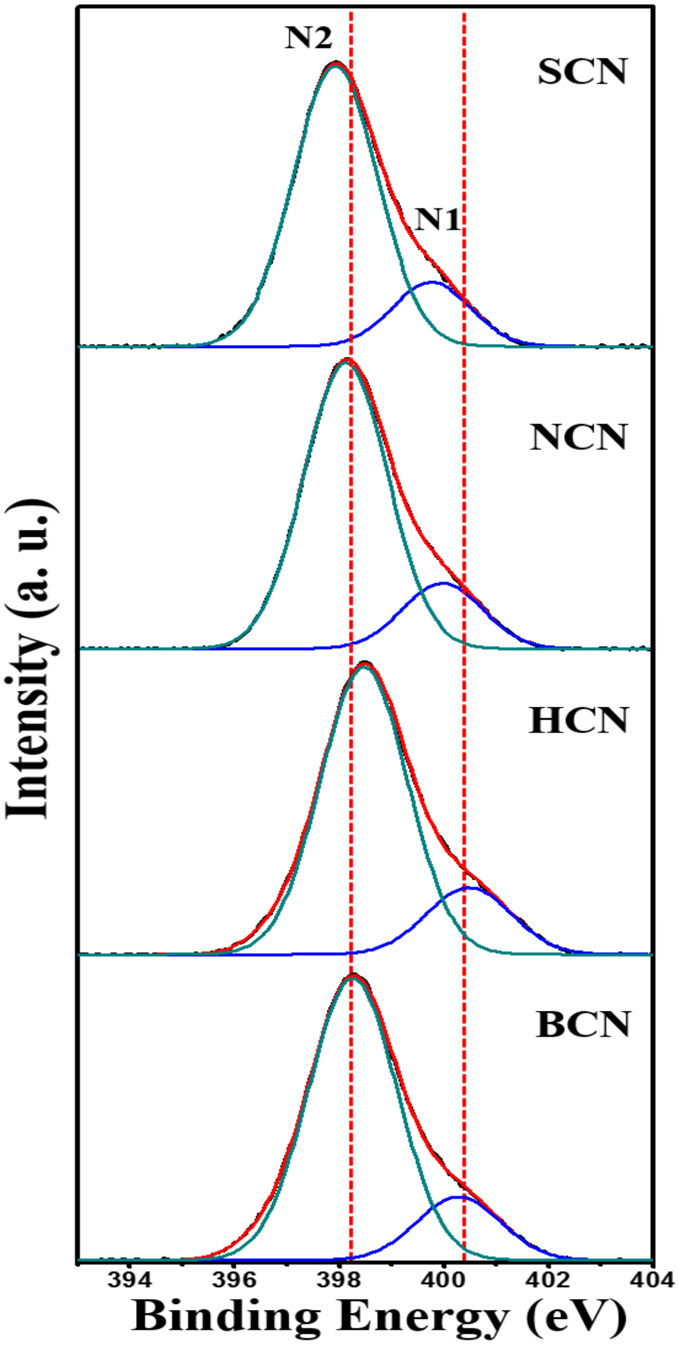
N2
N1
SCN
NCN
HCN
BCN
Intensity (a. u.)
394
396
398
400
402
404
Binding Energy (eV)

Figure S3C

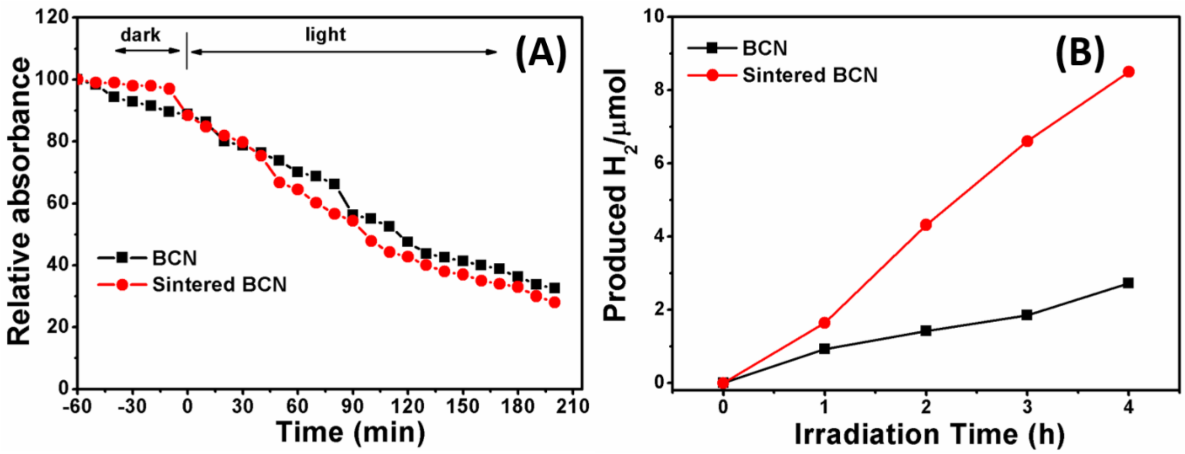

Figure S4 (A and B)

	**BCN**	**HCN**	**NCN**	**SCN**
**C conc. %**	**46.05**	**49.85**	**50.08**	**66.35**
**N conc. %**	**49.53**	**50.14**	**49.90**	**33.64**
**C/N ratio**	**0.93**	**0.99**	**1.01**	**1.97**

Table TS1

$g\text{-}C_3N_4$ Morphology	Sacrificial agent	Wavelength (nm)	Lamp power (W)	Hydrogen evolution	Reference
Lamellar structure	TEOA	> 420 nm	300	16.4 μmol h^{-1}	S1
Bulk $g\text{-}C_3N_4$	Methanol	> 395 nm	300	23 μmol g^{-1}	S2
Bulk $g\text{-}C_3N_4$	TEOA	Not provided	300	14 μmol h^{-1}	S3
graphite-like $g\text{-}C_3N_4$	Methanol	> 240 nm	Not provided	9.43 μmol h^{-1}	S4
Bulk $g\text{-}C_3N_4$	Methanol	> 400 nm	300	0.146 μmol h^{-1}	S5
$g\text{-}C_3N_4$ sheets	Methanol	~ 420 nm	3/80 (UV LEDs)	< 5 μmol g^{-1} h^{-1}	S6
$g\text{-}C_3N_4$ sheets	Methanol	> 400 nm	300	1.89 μmol h^{-1}	S7
Alkalinized $g\text{-}C_3N_4$	TEOA	> 400 nm	300	151 μmol h^{-1}	S8
Mesoporous $g\text{-}C_3N_4$	TEOA	> 400 nm	300	107 μmol h^{-1}	S9
BCN	TEOA	> 400 nm	300	1.26 μmol g^{-1}	Present work
HCN				5.54 μmol g^{-1}	
NCN				6.80 μmol g^{-1}	
SCN				34.00 μmol g^{-1}	

Table TS2

Supporting references:

S1. Chen, Y. et al. Origin of the enhanced visible-light photocatalytic activity of CNT modified g-C_3N_4 for H_2 production. Physical Chemistry Chemical Physics **16,** 8106-8113, doi:10.1039/C3CP55191A (2014).

S2. Suryawanshi, A. et al. Doubling of photocatalytic H_2 evolution from g-C_3N_4 via its nanocomposite formation with multiwall carbon nanotubes: Electronic and morphological effects. International Journal of Hydrogen Energy **37,** 9584-9589, doi:http://dx.doi.org/10.1016/j.ijhydene.2012.03.123 (2012).

S3. Zhang, G. et al. Iodine modified carbon nitride semiconductors as visible light photocatalysts for hydrogen evolution. Advanced Materials **26,** 805-809, doi:10.1002/adma.201303611 (2014).

S4. Wang, J., Huang, J., Xie, H. & Qu, A. Synthesis of g-C_3N_4/TiO_2 with enhanced photocatalytic activity for H2 evolution by a simple method. International Journal of Hydrogen Energy **39,** 6354-6363, doi:http://dx.doi.org/10.1016/j.ijhydene.2014.02.020 (2014).

S5. Ge, L. & Han, C. Synthesis of MWNTs/g-C_3N_4 composite photocatalysts with efficient visible light photocatalytic hydrogen evolution activity. Applied Catalysis B: Environmental **117–118,** 268-274, doi:http://dx.doi.org/10.1016/j.apcatb.2012.01.021 (2012).

S6. Shi, F. et al. ZnS microsphere/g-C_3N_4 nanocomposite photo-catalyst with greatly enhanced visible light performance for hydrogen evolution: synthesis and synergistic mechanism study. RSC Advances **4,** 62223-62229, doi:10.1039/C4RA11740A (2014).

S7. Ge, L. et al. Synthesis and efficient visible light photocatalytic hydrogen evolution of polymeric g-C_3N_4 Coupled with CdS quantum dots. The Journal of Physical Chemistry C **116,** 13708-13714, doi:10.1021/jp3041692 (2012).

S8. He, F. et al. The facile synthesis of mesoporous g-C_3N_4 with highly enhanced photocatalytic H_2 evolution performance. Chemical Communications **51,** 16244-16246, doi:10.1039/C5CC06713H (2015).

S9. Li, Y. et al. In situ surface alkalinized g-C_3N_4 toward enhancement of photocatalytic H_2 evolution under visible-light irradiation. Journal of Materials Chemistry A **4,** 2943-2950, doi:10.1039/C5TA05128B (2016).
